# 3D-CTBA及3D-VATS单操作孔行解剖性肺段切除治疗非小细胞肺癌

**DOI:** 10.3779/j.issn.1009-3419.2017.09.02

**Published:** 2017-09-20

**Authors:** 晓伟 佘, 云斌 顾, 春 徐, 心雨 宋, 畅 李, 成 丁, 俊 陈, 永生 龚, 军 赵

**Affiliations:** 1 215006 苏州，苏州大学附属第一人民医院胸外科 Department of Thoracic Surgery, the First Affiliated Hospital of Soochow University, Suzhou 215006, China; 2 215008 苏州，南京医科大学附属苏州市立医院北区胸外科 Department of Thoracic Surgery, Suzhou Municipal Hospital North District, Nanjing Medical University, Suzhou 215008, China; 3 215006 苏州，苏州大学附属第一人民医院放射科 Department of Radiology, the First Affiliated Hospital of Soochow University, Suzhou 215006, China

**Keywords:** 3D-CT, 支气管血管成像, 三维胸腔镜, 肺段切除术, 肺肿瘤, Three-dimensional computed tomography, Bronchography and angiography, Three-dimensional VATS, Segmentectomy, Lung neoplasms

## Abstract

**背景与目的:**

中国是肺癌高发地区，其发病率及死亡率在恶性肿瘤中均占首位。目前低剂量CT检查的普及使早期肺癌检出率显著提高，解剖性肺段切除目前广泛应用于Ia期非小细胞肺癌（non-small cell lung cancer, NSCLC）及不能耐受肺叶切除肺癌患者。但因肺段解剖结构及手术操作相对复杂，使得其具有较高的手术风险与难度。我们应用三维计算机断层扫描支气管血管成像（three-dimensional computed tomography bronchography and angiography, 3D-CTBA）及三维电视辅助胸部外科技术（three-dimensional video-assisted thoracic surgery, 3D-VATS）单操作孔行解剖性肺段切除微创手术技术治疗NSCLC，以探讨其临床效果，为其临床应用提供相关可行性及理论依据。

**方法:**

回顾性分析苏州大学附属第一人民医院胸外科2015年10月-2017年04月共施行57例术前对肺部病灶予以3D-CTBA重建以及术中应用3D-VATS单操作孔进行解剖性肺段切除治疗NSCLC病例。

**结果:**

全组均全腔镜下顺利完成，无中转开胸。手术时间平均（142.2±28.3）min，术中出血量平均（93.8±46.5）mL。平均淋巴结清扫数目（9.1±2.2）个，术后胸腔引流量平均（429.8±181.2）mL。术后留置胸管时间（2.8±1.1）d。平均住院时间（5.2±1.3）d。术后病理示良性病变9例，约占15.7%，恶性病变48例，约占84.2%。术后并发症：肺部感染3例（5.2%），肺不张1例（1.7%），少量咯血1例（1.7%），肺漏气2例（> 3 d, 3.5%），心律失常4例（7.0%）。术后平均随访10个月，无支气管胸膜瘘、乳糜胸、包裹性胸腔积液等并发症，随访患者中无复发及远处转移病例。

**结论:**

应用3D-CTBA及3D-VATS单操作孔行解剖性肺段切除治疗NSCLC的安全有效，适用于早期NSCLC以及不能耐受肺叶切除患者。

## Abstract (en)

**Background and objective:**

China is a high-incidence area of lung cancer, and its morbidity and mortality were the highest in malignant tumors. At present, the popularization of low-dose computed tomography (CT) examination has significantly improved the detection rate of early lung cancer, anatomical segmentectomy is currently widely used early in non-small cell lung cancer (NSCLC) and those who cannot tolerate lobectomy in patients with lung cancer. However, the anatomical structure of segment and its surgical operation is relatively complex, lead to segmentectomy has a high risk and difficulty. We performed anatomical segmentectomy by use of combining three-dimensional computed tomography bronchography and angiography (3D-CTBA) and three-dimensional video-assisted thoracic surgery (3D-VATS) single-operation-hole minimally invasive surgery in the treatment of NSCLC to investigate its clinical effect, and evaluate its clinical relevant feasibility and theoretical basis.

**Methods:**

We carried out a retrospective review of the 57 cases by use of combining 3D-CTA in preoperative and 3D-VATS single-operation-hole anatomical segmentectomy in intraoperative in the treatment of NSCLC performed in Department of Thoracic Surgery, the First Affiliated Hospital of Soochow University from October 2015 to April 2017.

**Results:**

The whole group was successfully performed by use of VATS without anyone conversion to thoracotomy. The mean operation time was (142.2±28.3) min, and the mean blood loss was (93.8±46.5) mL. The mean number of lymph node dissection was (9.1±2.2), and the mean postoperative thoracic drainage was (429.8±181.2) mL. The postoperative retention of chest tube time was (2.8±1.1) d. The mean hospitalization time was (5.2±1.3) d. The postoperative pathology showed 9 cases with benign lesions, accounting for 15.7%, 48 cases with malignant lesions, accounting for 84.2%. Postoperative complications: pulmonary infection in 3 cases (5.2%), atelectasis in 1 case (1.9%), small amount of hemoptysis in 1 case (1.9%), lung leakage > 3 d in 2 cases (3.5%). Arrhythmia in 4 cases (7.0%). The patients were followed up for 10 months. No complications occurred such as bronchial pleural fistula, chylothorax, encapsulated pleural effusion and those patients were followed up without recurrence and distant metastasis.

**Conclusion:**

The use of combining 3D-CTBA and 3D-VATS single-operation-hole to anatomical segmentectomy is safe and effective in the treatment of NSCLC and is suitable for early non-small cell lung cancer, especially those can not tolerate lobectomy.

中国是肺癌高发地区，其发病率及死亡率在恶性肿瘤中均占首位^[1]^。目前低剂量计算机断层扫描（computed tomography, CT）检查的普及使早期肺癌检出率显著提高，解剖性肺段切除适用于Ia期非小细胞肺癌（non-small cell lung cancer, NSCLC），同时解剖性肺段切除亦可用于不适于行肺叶切除患者，因而使其在治疗NSCLC得到较为广泛的应用^[2, 3]^。但肺段解剖结构及手术操作相对复杂，因此解剖性肺段切除具有较高的手术风险与难度。我们通过术前应用三维计算机断层扫描血管成像（three dimensional computed tomography bronchography and angiography, 3D-CTBA），可精确定位肿块位置，分析其与段支气管、动静脉以及临近组织结构的关系^[4]^。同时术中应用三维电视辅助胸部外科技术（three dimensional video-assisted thoracic surgery, 3D-VATS）进行手术，以进一步利于手术操作，减少手术风险及术中出血，缩短手术时间。我科2015年10月-2017年4月共施行57例3D-CTBA及3D-VAST单操作孔解剖性肺段切除治疗NSCLC，现对其进行回顾性分析与总结，为其临床应用提供相关可行性及理论依据。

## 1 材料与方法

### 1.1 患者资料

患者共57例，男性24例，女性33例。平均年龄（59.2±11.8）岁。术前由胸部CT明确诊断，对小于8 mm病灶予以定期观察，对≥8 mm、≤20 mm的结节根据其CT影像学特征或观察1个月-3个月后无明显变化者方考虑手术。术前评估心肺功能，行头颅磁共振成像（magnetic resonance imaging, MRI）、腹部B超及骨扫描检查排除手术禁忌。根据国内外相关文献报道^[5-7]^及结合我科经验总结，选择肺段手术标准：①单纯原位腺癌或微浸润腺癌且直径≤2 cm周围型病灶；②结节磨玻璃密度（ground-glass opacity, GGO）成分≥50%且结节倍增时间≥400 d；③性质难于明确位置较深的病灶；④肺内多发病变需联合切除或孤立性肺转移瘤；⑤妥协性肺段切除术，即心肺功能较差的高龄患者或无法耐受肺叶切除的患者。

### 1.2 手术方法

对所有患者术前应用Deep Insight软件行3D-CTBA成像重建，以明确肿块所处肺段。根据3D-CTBA图像，分析段动静脉靶血管及段支气管位置、结构关系，预先规划手术方式。对基底段各分段切除及两肺上叶各段等因其结构相对复杂，术前应该充分评估及设计手术方案。对病灶较小病例，术前通过CT定位置入导引钢丝（Hook wire）于病灶毗邻部位，以便术中定位。

患者全身麻醉，双腔管通气，健侧90°卧位。采用单操作孔手术方式：即取第7或8肋间腋中线1 cm为腔镜孔，腋前线第4（结节位于上、中叶）或第5（结节位于下叶）肋间取3 cm切口为操作孔。术者位于患者腹侧，助手于对侧。胸腔镜设备采用德国KARL STORZ 3D胸腔镜系统。术者及助手佩戴3D偏振眼镜，显示器显示三维立体图像。根据术前3D-CTBA成像预先设计的方案，结合术中三维立体成像具体情况进行操作（图 1）。对结节位置较深并贴近段血管、段气管者，直接行肺段切除；对结节离肺表面浅表者，先予以行楔形切除送快速病理。对叶间裂发育良好的病例从叶间裂开始处理血管。若叶间裂发育不全，则于肺门处入路。仔细辨别靶段动静脉，尤其对术前根据3D-CTBA成像评估存在解剖结构变异者，应该小心避免误伤其他肺段血管、支气管。对肺段细小动、静脉分支，可以电凝或超声刀切断，较大的分支可以采用结扎或腔镜直线切割缝合器离断。常规采用“肺膨胀-萎陷法”明确肺段交界^[8]^。即夹闭靶段支气管后张肺，明确靶段支气管正确；切断靶段支气管后张肺，待靶段肺组织膨胀后单肺通气。约15 min左右后余肺段萎险。根据充气的靶段肺组织与萎陷的肺组织之间形成界限组合使用电刀、超声刀和直线切割缝合器离断段间交界面。对恶性结节所有手术切缘必须保证≥2 cm、或至少等于肿块直径，必要时行联合肺段切除。常规行N_1_（10组-13组）及N_2_淋巴结采样活检，阳性者改肺叶切除+系统性淋巴结清扫。

**1:**
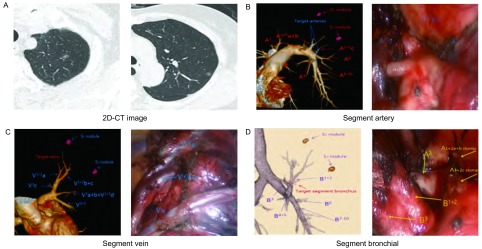
术前三维重建与术中操作。A：2D-CT平扫示左肺尖后段见两结节；B：术前3D-CTBA肺段动脉重建并与术中手术操作对照；C：术前3D-CTBA肺段静脉重建并与术中手术操作对照；D：术前3D-CTBA肺段支气管重建并与术中手术操作对照。术中视频图片由3D转换为2D图片；图中缩写：A为动脉，V为静脉，B为支气管，S为肺段。 Preoperative three-dimensional reconstruction and intraoperative operation. A: 2D-CT scan showed two nodules in the segmentum apicoposterius of the left lung; B: preoperative 3D-CTBA segment artery reconstruction and contrasted with intraoperative operation; C: preoperative 3D-CTBA segment vein reconstruction and contrasted with intraoperative operation; D: preoperative 3D-CTBA segment bronchial reconstruction and contrasted with intraoperative operation. The image of video in the operation is converted to 2D images from 3D; abbreviation in the figure: A: artery; V: vein; B: bronchial; S: segment.

## 2 结果

全组均全腔镜下顺利完成，无中转开胸，手术切除肺段部位及例数见表 1。手术时间平均（142.2±28.3）min，术中出血量平均约为（93.8±46.5）mL。平均淋巴结清扫数目（9.1±2.2）个，术后胸腔引流量平均约（429.8±181.2）mL。术后留置胸管时间（2.8±1.1）d。平均住院时间（5.2±1.3）d。术后并发症：肺部感染3例（5.2%），肺不张1例（1.7%），少量咯血1例（1.7%），肺漏气2例（> 3 d, 3.5%）。心律失常4例（7.0%）。术后病理示良性病变9例，约占（15.7%），恶性病变48例，约占（84.2%），详见表 2。术后平均随访10个月，无支气管胸膜瘘、乳糜胸、包裹性胸腔积液等并发症，随访恶性结节患者中无复发及远处转移病例。

**1:** 手术切除肺段部位及例数 The segmentectomy position and the number of cases

Segmentectomy position	Left lung (*n*=26)	Right lung (*n*=31)
S1	-	3
S2	-	2
S3	2	6
S1+S2	3	4
S1+S2+S3	4	-
S4+S5	5	-
S6	8	10
S7+S8	1	2
S9+S10	1	1
S7+S8+S9+S10	2	3

**2:** 术后病理及例数 the postoperative pathology and the number of cases

Pathological category	Number of cases [*n* (%)]
Benign	9 (15.7)
Benign nodule	3 (5.2)
Atypical adenomatous hyperplasia	6 (10.5)
Malignant	48 (84.2)
Adenocarcinoma *in situ*	25 (43.8)
Minimally invasive adenocarcinoma	19 (33.3)
Other types and metastase tumor	4 (7.0)

## 3 讨论

随着低剂量CT平扫的在我国的普及，使得肺癌的检出率显著提高，尤其是对于早期肺癌的诊断^[9]^。目前针对早期肺癌的治疗的手段，由原先标准的解剖性肺叶切除逐渐转向解剖性肺段切除。解剖性肺段切除目前成为治疗早期肺癌的热点，但也存在一定争论，焦点在于解剖性肺段切除是否与肺叶切除达到相等的肿瘤学治疗效应。目前相关研究表明，对于肿瘤≤2 cm的Ia期NSCLC，行解剖性肺段切除，其术后完全生存率与行肺叶切除术无差异，尤其是病理分期原位癌及微浸润性腺癌行肺段切除与肺叶切除远期生存无明显差异^[10, 11]^。我们对诊断早期肺癌患者拟实施肺段切除亦进行严格选择，除妥协性解剖肺段切除，对周围直径≥2 cm病灶以及对术中病理提示浸润性腺癌或淋巴结阳性患者，常规行肺叶切除及系统性淋巴结清扫。

相比肺叶切除而言，对Ia期肺癌患者予肺段切除一方面进行了解剖性病灶肺切除，同时也可最大限度的保留健康肺组织，从而使患者术后获得更好的生存质量。尤其对高龄、肺功能较差患者，妥协性解剖肺段切除可使患者获得最大收益，这也是肺段切除在治疗NSCLC得以应用的主要因素之一。但对比肺叶切除术，肺段切除由于解剖结构更为复杂，肺段动静脉血管更易出现变异，且对血管及段支气管的走行需要有一定的空间立体想象感，因而肺段手术需要更高的手术操作能力以及更高的学习曲线。普通薄层CT扫描因二维显示图像有时往往难以明确结节所在肺段以及靶段动静脉走向，尤其是在邻近段间交界处，而对肺段的动静脉出现的解剖结构变异术前则往往难以明确。3D-CTBA可明确结节所在肺段区域，并可立体显示靶血管的三维走向，在术前即能明确靶血管所存在血管解剖结构变异，因此对术前手术操作方式及程序的选择与设计提供较大帮助。我们通过术前对靶结节及血管、支气管行三维重建，尤其针对两肺上叶及下肺基底各段行重建，术前制定良好手术设计方案以避免术中误操作损伤正常紧邻段血管及支气管。

电视辅助胸部外科技术（video-assisted thoracic surgery, VATS）的出现与革新极大改变胸部手术的治疗方式。由于视频成像系统、内窥镜、内镜切割吻合器及专用手术器械的技术进步，从而使VAST成为肺部肿瘤手术治疗的有效方式。与其他许多外科微创手术领域相类似，胸部微创手术技术已经发展成为使手术并发症最小化、更少的疼痛、更好术后的生活质量以及更快术后康复的有效胸部外科治疗手段，并具有更好的美容效果和更好的性价比。从目前观察的结果来看，胸部微创手术远期疗效至少相当于传统开放手术，从而成为治疗高风险胸部手术患者的有效替代方案^[12]^。目前胸腔镜技术由原先的4孔、3孔逐渐向更小创伤的2孔及单孔发展，我们采用单操作孔行解剖性肺段手术操作，以减少患者肋间神经的损伤以及手术创伤，减轻术后疼痛，利于患者术后快速康复。

但目前2D胸腔镜因显示二维手术操作图像，从而导致手术操作立体感及纵深感的缺乏。对操作者而言往往存在想法与操作的“失真”与不协调，尤其对于初学者而言。3D胸腔镜可以将目标最大放20倍，同时可呈现手术视野真实立体感，使人与手术器械更加协调，操作感贴近传统开胸，因而可使手术操作更加自然流畅，并可减少初学者腔镜学习曲线。其他相关学科研究及实验也显示，3D腔镜较2D相比可明显减少术中错误、缩短手术时间以及更少的出血，显著降低腔镜初学者学习曲线^[13]^。相对于肺叶解剖，肺段其解剖结构更为复杂，也较易出现解剖结构的变异，同时其呈现更多的立体解剖结构。我们在术前应用3D-CTBA技术，可使得术前在脑海中形成该肺段的一定的三维立体解剖，辨别其所可能出现的解剖结构异常，从而较好的规划出手术预定方案与流程。通过术中应用3D-VAST技术，我们将预定的手术设想通过三维立体手术操作视野进行转化，从而使得手术流程变得简洁流畅，手术操作更加精准。同时根据术前预定手术操作方案以所评估解剖结构变异，从而降低手术相关误操作及损伤，术中出血亦相应减少。

解剖性肺段切除术后咯血的因素主要为：①损伤段间静脉，尤其在分离段间平面时易发生。②误断保留肺段的静脉，导致保留肺段血液回流受阻，称为“静脉梗死”^[8]^。我们通过术前及术后的3D精准预判及手术操作，显著减少误断保留肺段的静脉以及段间平面段间静脉的损伤几率，从而减少术后咯血的发生率。肺段切除术后出现肺漏气我们分析除了闭合断端手术技巧的原因，主要因素是由于肺段间交界处无明显的标志，导致离断面闭合不佳致漏气。术前3D-CTBA成像技术可明确肿块所在肺段以及其支气管、段支气管走向，并可预先判断肺段范围，通过术中应用膨肺及萎险肺技术可较完好地切除肺段范围，同时可减少术后肺扭转等并发症。

通过3D-CTBA及3D-VAST两项技术的结合以及单操控孔微创技术的应用，使我们在术前更好的进行了手术操作方案的设计以及风险预案的评估，术中更好的进行精准的手术操作。通过对合适的病例进行单操作孔肺段切除治疗，从而使患者获得了良好的治疗效果，值得在临床加以推广与应用。
